# The application of system dynamics modelling to environmental health decision-making and policy - a scoping review

**DOI:** 10.1186/s12889-018-5318-8

**Published:** 2018-03-27

**Authors:** Danielle J. Currie, Carl Smith, Paul Jagals

**Affiliations:** 1 School of Public Health, The University of Queensland, Herston, Brisbane, QLD 4006 Australia; 2 School of Business, The University of Queensland, St. Lucia, Brisbane, QLD 4072 Australia; 3 Child Health Research Centre, The University of Queensland, South Brisbane, QLD 4101 Australia

**Keywords:** System dynamics modelling, Environmental health, Decision support systems, Decision-making, Policy, Scoping review

## Abstract

**Background:**

Policy and decision-making processes are routinely challenged by the complex and dynamic nature of environmental health problems. System dynamics modelling has demonstrated considerable value across a number of different fields to help decision-makers understand and predict the dynamic behaviour of complex systems in support the development of effective policy actions. In this scoping review we investigate if, and in what contexts, system dynamics modelling is being used to inform policy or decision-making processes related to environmental health.

**Methods:**

Four electronic databases and the grey literature were systematically searched to identify studies that intersect the areas environmental health, system dynamics modelling, and decision-making. Studies identified in the initial screening were further screened for their contextual, methodological and application-related relevancy. Studies deemed ‘relevant’ or ‘highly relevant’ according to all three criteria were included in this review. Key themes related to the rationale, impact and limitation of using system dynamics in the context of environmental health decision-making and policy were analysed.

**Results:**

We identified a limited number of relevant studies (*n* = 15), two-thirds of which were conducted between 2011 and 2016. The majority of applications occurred in non-health related sectors (*n* = 9) including transportation, public utilities, water, housing, food, agriculture, and urban and regional planning. Applications were primarily targeted at micro-level (local, community or grassroots) decision-making processes (*n* = 9), with macro-level (national or international) decision-making to a lesser degree. There was significant heterogeneity in the stated rationales for using system dynamics and the intended impact of the system dynamics model on decision-making processes. A series of user-related, technical and application-related limitations and challenges were identified. None of the reported limitations or challenges appeared unique to the application of system dynamics within the context of environmental health problems, but rather to the use of system dynamics in general.

**Conclusions:**

This review reveals that while system dynamics modelling is increasingly being used to inform decision-making related to environmental health, applications are currently limited. Greater application of system dynamics within this context is needed before its benefits and limitations can be fully understood.

## Background

There is growing recognition of the complexity that underlies environmental health problems and their management [1] and with it the need for sound environmental health policy and decision-making that embraces this complexity [2]. To embrace this complexity, managers of environmental health issues must move away from reductionist approaches to decision-making, as environmental health hazards are influenced by a multitude of interacting factors that may not initially appear related [3]. This can result in policy resistance, which is the tendency for policies to be ineffective or have unintended consequences that create new problems or exacerbate the original problem [4]. Examples include the digging of wells in South East Asia to combat diarrhoea caused by poor quality surface water, resulting in the largest exposure in history of a population to arsenic-contaminated groundwater [5], and making dwellings in the United Kingdom more air tight to improve household energy efficiency, resulting in increased indoor exposure to radon [6].

Decision-makers, more than ever, are required to integrate scientific and expert-based evidence into decision-making. Complexity can hinder evidence-based decision-making by slowing and weakening the ways in which evidence is collected, interpreted and translated into action [7]. Evidence for complex problems can be lacking, limited and ambiguous, and is often presented without links to the broader context for which the evidence is required. Also, the analytical tools currently used to support evidence-based decision making, such as statistical modelling, are limited in their ability to explain causation and the effect of non-linear interactions and feedback on the behaviour of complex systems [8]. Given this, there is a need to provide decision-makers with the tools necessary to understand complex environmental health issues as part of implement more scientific decision-making processes.

System dynamics modelling is a problem-oriented modelling approach pioneered by Jay Forrester in the late 1950’s to help corporate managers better understand industrial problems [9]. Since then, its application has expanded to fields ranging from ecology to economics. Examples of its use include modelling the dynamics of the earth’s climate [10], healthcare systems [11], the food industry [12] and the military [13]. System dynamics involves causal mapping and the development of computer simulation to understand system behaviour. Policy and scenario options are then systematically tested to answer “what-if” questions [14]. This allows policy-makers to experiment with their decisions before implementation in the real world. This decision-experimentation creates a learning environment in which policy makers gain a better understanding of how the system will respond to their decisions and the potential unintended consequences of decisions.

Conventional problem solving tends to approach complex problems by breaking them into their component parts and examining them separately. System dynamics centres on the idea that problems exist due to the interactions, feedback loops and material and information delays among component parts within a system. System dynamics therefore focuses on the relationships between the parts rather than focusing on separate parts in isolation [15]. System dynamics also draws strongly on the concept of endogeneity, meaning that it seeks to find explanations for system behaviour by understanding the internal structure of a system rather than focusing on factors external to the system [16]. For example, when trying to understand why medical errors still occur in seemingly well-managed hospital settings, the temptation is to blame the problem on individual-level factors beyond the control of the hospital, such as incompetent or lazy practitioners. An endogenous perspective would take the view that most medical errors are the result of a combination of systemic factors such as the practitioner’s patient load, training, medication labelling practices, hours worked, etc. and thus the solution lies within understanding how these factors contribute to medical errors and modifying the system to prevent errors.

In this paper we review the application of system dynamics to the management of environmental health issues. System dynamics has been used to understand the interactions between human health and the environment before, such as zoonotic infections [17], the relationship between air pollution, health and population growth [18], the societal costs and benefits of commuter bicycling [19], the effect of land use and transport policies on health [20] and malaria control [21]. However, many of these applications do not demonstrate how, or if, an understanding of system dynamics can lead to better policy and decision-making. Therefore it remains unclear whether the findings of these previous applications of system dynamics have remained within the confines of academia or if they have been applied to inform real decision-making processes.

### Objective

An increasing number of authors advocate for the use of systems thinking and system dynamics in epidemiology, public health and health service delivery [22–24], but it is unclear to what extent system dynamics has actually been used to inform policy or decision-making processes in environmental health service delivery. The objective of this review was to answer the following question “How is system dynamics being used to inform decision-making processes related to environmental health?” To answer this question we conducted a scoping review of the scientific and the grey literature.

## Methods

We used a scoping review method because the objective of the review was to broadly explore how system dynamics has been used to inform environmental health decision-making processes, as opposed appraising the quality of individual studies. Scoping reviews search a wide range of research and non-research reports to uncover the breath and extent of research and application for a particular topic [25]. The design of this study was guided by the scoping review guidelines developed by Arksey and O’Malley [26] and the recommendations proposed by Levac, et al. [27], but included an additional step where papers where re-screened based on their relevancy to objectives of this review. The result was a five-step process: formulation of an objective, identification of potentially relevant science and grey literature through a systematic search, secondary screening using a framework of relevancy-based criteria to select literature the most suitable for review, recording of relevant key themes emerging from the literature, and reporting of results.

Given the extent of this scoping review, we do not claim that the literature captured is an exhaustive or complete list of the scientific and grey literature on this topic, rather a representative sample of the available literature.

### Identifying relevant reports

A systematic search of the literature in the Pubmed, Embase, Web of Science and Scopus databases was conducted. Following an initial broad search of the literature, and in consultation with a health-science librarian, three conceptual focus areas (search themes) with associated search terms were identified: environmental health, system dynamics, and decision-making. The search terms used in combination included (“environmental health” OR “environment and public health” OR “environment”) AND (“systems thinking” OR “systems science” OR “systems approach” OR “systems theory” OR “systems analysis” OR “system dynamics” OR “dynamic systems”) AND (“health policy” OR “policy making” OR “decision making” OR “health planning” OR “policy” OR “‘decision support system” OR “decision support techniques” OR “environmental policy”). Where available, we used indexing terminology (MeSH and Emtree) that corresponded to the search terms. The search was limited to reports published in English between the years 2000 and 2016. The review was limited to reports published after 2000 to reflect the significant increase in publications related to systems thinking and systems science that has occurred since then [28], as well as the rapidly-evolving nature of the field of environmental health [29].

The same search terms were used in the Google search engine to similarly identify relevant grey literature.

### Report selection

Reports captured though the search of the academic and grey literature were initially screened according to inclusion criteria developed for this review (Table 1).

**Table 1:** Inclusion criteria for initial screening process

Inclusion criteria	- Primary focus of study is examining an environmental health problem or topic - Reports on the original application of system dynamics methods (is not a review paper, position paper or commentary) - Demonstrates a link between system dynamics and a population-level policy or decision-making process - Published after January 1, 2000 - Written in English

To ensure that the retained reports would meet the objective of this review, we developed three relevancy criteria (Table 2) to determine the strength of each paper pertaining to the three conceptual focus areas of this review: environmental health, system dynamics and decision-making. This relevancy approach was adopted because the concepts of environmental health and decision-making are broadly-defined concepts that do not lend themselves to specific inclusion criteria. Reports retained during the initial screening process were then screened according to the relevancy criteria. Reports classed as ‘somewhat relevant’ or ‘not relevant’ to one or more of the relevancy criteria were excluded from further review.

**Table 2:** Relevancy Criteria

RC 1 - Subject / content relevancy – Area of environmental health service delivery	
*The report’s primary focus is related to the assessment and control of environmental factors:*	Highly Relevant – that *directly* contribute to the creation of health-supportive environments, but where the protection or promotion of human health and wellbeing is *directly* included in the goal of the investigation.
	Relevant - that *directly* contribute to the creation of health-supportive environments, but where the protection or promotion of human health and wellbeing is *not directly* included in the goal of the investigation.
	Somewhat Relevant - that *indirectly* contribute to the creation of health-supportive environments, and where the protection or promotion of human health and wellbeing is *not directly* included in the goal of the investigation.
	Not Relevant - that do not affect human health or the creation of health-supportive environments.
RC 2 - Methodological Relevancy - Use of system dynamics to address an environmental health problem	
*The report describes the use of system dynamics to examine the assessment or control of one or more environmental factors:*	Highly Relevant - *directly* in relation to the impact that the factor can potentially have on population health.
	Relevant - *indirectly* in relation to the impact that the factor(s) can potentially have on human health. This includes reports that include environmental health hazard or risk factors in the system dynamics modelling without directly addressing their impact on population health.
	Somewhat Relevant - which can reasonably be assumed is done, at least in part, for the protection of population health or the creation of health-supportive environments, but does *not directly or indirectly* examine the impact that the factor can potentially have on population health.
	Not Relevant - not in relation to the impact that the factor can potentially have on human health.
RC 3 - Application Relevancy – Application in public decision-making	
*The report outlines the use of system dynamics modelling in a way that is intentionally intended:*	Highly Relevant – to inform a *specific* policy or decision-making processes including *specifying* how the modelling process or model findings we used to inform the policy or decision-making process.
	Relevant – to inform a *specific* policy or decision-making processes *without specifying* how the modelling process or model findings we used to inform the policy or decision-making process.
	Somewhat Relevant - to inform *non-specific* policy or decision-making processes, *without specifying* if or how the modelling process or model findings were used to inform a policy or decision-making process.
	Not Relevant - not in a way that is intentionally intended to inform policy or decision-making processes

Reports that were classed ‘relevant’ or ‘highly relevant’ for all three relevancy criteria were included in this review. For each retained report, data on the location, sector of application, project objectives, link to health and type of system dynamics analysis techniques used was organised in a Microsoft Excel® spreadsheet. Reports were also analysed to identify key themes related to the stated rationales for using system dynamics, the intended impact of system dynamics modelling on decision-making, the targeted scale of decision-making and limitations and challenges associated with system dynamics modelling.

## Results

### Search findings

**Fig. 1:**
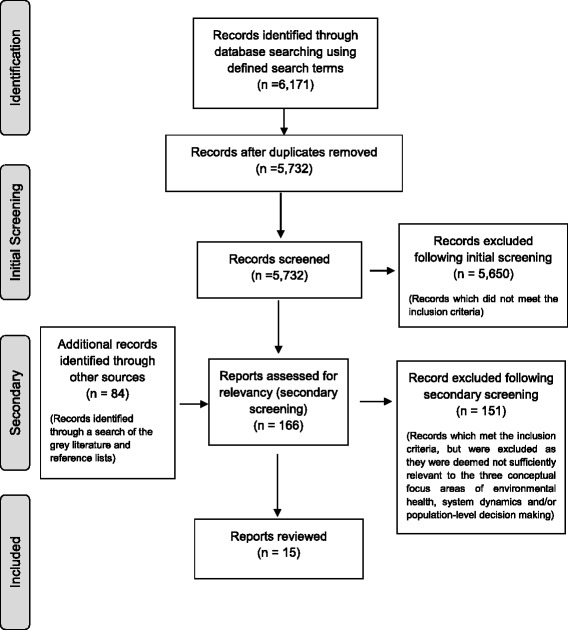
Selection process and results

A total of 5732 unique reports were identified by searching scientific databases (Fig. 1). An additional 84 publications were identified through a search of the grey literature, a search of the reference lists of retained articles, and the ‘related articles’ or ‘cited by’ section in the databases. At the end of initial screening using the inclusion criteria, 166 reports were retained. Screening according to the relevancy criteria left 15 reports for final review.

### General characteristics

The general characteristics of the 15 reviewed reports are outlined in Table 3. Of these, 47% (*n* = 7) of the studies were conducted in the United States of America. The rest were conducted in the United Kingdom (*n* = 2), Canada (*n* = 1), New Zealand (n = 1), Taiwan (n = 1), Colombia (n = 1), Bolivia (n = 1) and in both Ghana and Ethiopia (n = 1). The majority of the studies (67%) were conducted within the last 5 years (2011–2016), which suggests recent and increasing interest that systems science that has been identified in other areas of the published health literature [28].

**Table 3:** General characteristics of selected studies

Author, year	Location	Sector	Study/Project Objective	Health Link	Use of System Dynamics
Brennan, et al., 2015	United States of America	Health	identify trends and underlying feedback systems hypothesized by stakeholders as driving local change in health behaviours and obesity	Explicit	Systemic analysis
Chen, et al., 2005	Taiwan	Urban and Regional Planning	develop a dynamic strategy planning theory and system for sustainable river basin land use management	Inferred	Systemic analysis, simulation and policy analysis
Feola, et al., 2012	Colombia	Agriculture	uncover the social processes underlying the misuse of personal protective equipment, and support the identification and evaluation of intervention strategies	Explicit	Systemic analysis, simulation and policy analysis
Kenealy, et al., 2012,	New Zealand	Health	assess the usefulness of a national and a local system dynamics model of cardiovascular disease to planning and funding decision makers	Explicit	Systemic analysis, simulation and policy analysis
Kolling, et al., 2016	United States of America	Transportation	design an approach that uses dynamics systems modelling to explore the interplay among actions and decisions that lead to healthier and more sustainable communities	Explicit	Systemic analysis, simulation and policy analysis
Lane, 2014	United Kingdom	Food	investigate the relative significance of the foodborne transmission mechanisms on the scale of norovirus outbreaks and identify intervention leverage points	Explicit	Systemic analysis, simulation and policy analysis
Loyo et al., 2013	United States of America	Health	align stakeholders to develop a comprehensive strategy for reducing chronic diseases and related costs	Explicit	Systemic analysis, simulation and policy analysis
Macmillan et al., 2016	United Kingdom	Housing	develop a collaborative understanding of the complex system linking housing, energy and wellbeing	Explicit	Systemic analysis
Mahamoud, Roche and Homer, 2013	Canada	Health	investigate causal pathways between population health risk factors and health outcomes and identify policy options related to the social determinants of health	Explicit	Systemic analysis, simulation and policy analysis
Newman, et al., 2003	Bolivia	Health	develop models that explicitly link policy actions with results in the context of malaria control	Explicit	Systemic analysis, simulation and policy analysis
Olabisi, et al. 2012	United States of America	Health	develop a tool that is useful for local decision-makers responding to extreme heat events	Explicit	Systemic analysis, simulation and policy analysis
Pasqualini et al., 2006	United States of America	Public Utilities	investigate the consequences of disruptions in potable water distribution systems	Explicit	Systemic analysis and simulation
Raschid-Sally, et al., 2013	Ghana, Ethiopia	Water	examine the impacts of climatic and demographic changes on urban water resources management and develop a strategic action plan based on improved water resource management	Inferred	Systemic analysis, simulation and policy analysis
Stave and Dwyer, 2006	United States of America	Urban and Regional Planning, Transportation	improve the ability of local agencies and government entities to integrate land use, air quality and transportation planning	Inferred	Systemic analysis, simulation and policy analysis
Stave, 2002	United States of America	Transportation	develop policy recommendations to address traffic congestion and regional air quality problems	Inferred	Systemic analysis, simulation and policy analysis

The reports used system dynamics to inform decision-making within, and across, a number of different sectors, and all relevant to environmental health. The greatest number came from within the health sector (*n* = 6), with the remaining coming from the transport sector (*n* = 2), the public utilities sector (*n* = 1), the water sector (n = 1), the housing sector (n = 1), food sector (n = 1), the agricultural sector (n = 1), and the urban and regional planning sector (n = 1). Additionally, one report was multi-sectoral within both the urban and regional planning and the transportation sector.

The majority of reports (*n* = 11) explicitly dealt with the relationship between environment and human health, using system dynamics to map and/or model system structures and behaviours that govern environmental conditions and their effect on human health. The remainder of reports (*n* = 4) had an inferred link to health. In these cases, structural and behavioural links between environmental conditions and environmental health hazards, such as air pollution and water pollution, were modelled. These models were broadly focused on creating and testing policies whose multiple objectives included the protection of human health or the creation of health-supportive environments.

The majority of reports (*n* = 13) used system dynamics to conduct systemic analysis of a problem and then used simulation and policy analysis to model and test the outcome of various policies. The remaining two reports used system dynamics in only a qualitative way, using causal loop diagrams to qualitatively analyse the relationship between system structure and problem behaviours.

### Stated rationales for using system dynamics

Eleven of the publications stated one or more rationales for using system dynamics methods. Many of the stated rationale focused on the technical capabilities of system dynamics models, including their ability to identify and account for sources of uncertainty in the understanding of causal relationships [30, 31], account for long time-delays between action and effect [30], reveal the endogenous sources of system behaviour [32, 33], and link the dynamic effects of policy actions to system structure and behaviour [31, 34, 35]. Others focused on the predictive capabilities of system dynamics models including their ability to integrating multiple types of information into policy analysis [36, 37], assess program and policy intervention trade-offs [38, 39], and explore the effects of proposed policies over a chosen time scale [35, 38]. Lastly, a number of the publications also included process-related factors in their rationale for using system dynamics modelling, including its strength at making sense of the complexity of the problem [33, 38], and its potential to facilitate inter-sectoral and stakeholder engagement [33, 37–39].

### Intended impact of system dynamics modelling on decision-making

The intended users of the outcomes of system dynamics modelling fell into two categories: the decision- or policy-makers themselves, and the stakeholders affected by or involved in decision-making processes (Table 4).

**Table 4:** Intended impact of system dynamics on decision-making processes

Intended impact	Number of studies [reference]
Direct decision-maker decision-support	
Providing specific policy analysis related to an existing or anticipated policy issue	4 [31, 32, 40, 43]
Providing continuous use as a tool integrated into existing or future decision-making processes	3 [41, 42, 53]
Indirect decision-maker decision-support	
Educating decision-makers about system structure and behaviour	5 [30, 34, 36, 40, 42]
Confirming or challenging decision-makers’ beliefs or understanding of the problem	3 [34, 36, 42]
Facilitating inter-sectoral planning and decision-making processes	4 [30, 36, 40, 42]
supporting advocacy for a particular decision	2 [30, 42]
Stakeholder engagement and decision-support	
Helping stakeholders and decision-makers develop and articulate a shared understanding of the problem	7 [33, 35–40]
Facilitating the inclusion of stakeholder perspective in policy analysis process	6 [35–40]
Communicating information about the problem and policy options to stakeholder	1 [36]
Serving to as a catalyst for stakeholder action	1 [39]
Promoting stakeholder buy-in to policy recommendations	2 [33, 40]

One of the key uses of system dynamics identified in the reports was decision support for making policies and decisions. In these instances, the goal of the system dynamics modelling process was to compare the results of various policy scenarios to inform strategic policy decisions. Some of the models were designed to provide decision-support for a specific project or policy issue, such as the model designed in Kolling et al. [40], which was used to explore the community-level impacts and viability of a light rail project. Other models were intended for integration into existing or future decision-making processes, such as the model described in Chen et al. [41], which was designed for the model to be integrated directly into the analysis and review phases of a county-level urban planning process.

System dynamics models were also used to influence the way that decision- or policy-makers approach a particular topic or policy issue. In several of these reports the authors describe the modelling process itself as having significant benefit, irrespective of the results the model produced. Stave and Dwyer [42] reported that the collaborative process of creating the system dynamics model provided a means for integrating traditionally separate land use, transportation and air quality planning processes. This integration compelled decision-makers in each sector to shift their perspective away from focusing solely on their particular area of practice to viewing the system as a whole.

This review found that applications of system dynamics to inform or influence environmental health decision-making extended beyond those targeted directly at informing decision-makers, and also included processes targeted at engaging stakeholders in decision-making processes. For example, Stave [37] used system dynamics modelling as a means to integrate stakeholder feedback into the decision-making process, with the idea that if stakeholders are involved in making strategic decisions, they are more likely to ultimately help implement the outcome of those decisions.

### Targeted scale of decision-making

The review also showed that system dynamics was used to inform policy and decision making processes at two distinct levels: macro-level decision making (national or international) and the micro-level (local, community or grassroots).

System dynamics was used to exclusively support micro-level decision-making processes in over half of the studies (*n* = 9). Macro-level policy and decision-making was the primary focus of four studies targeted at decision-making in federal/national government departments.

Two studies used system dynamics to influence decision-making at both levels. The URAdapt project [43] targeted decision-making processes and policy actors at both the local and national level because the urban water supply systems in Addis Ababa and Accra fell under both the jurisdiction of local municipalities and national-level government departments. System dynamics was used in the Durham-Orange Light Rail Project in Durham, North Carolina [40] to inform and engage both federal and local decision-makers because components of the project fell under mandates at both levels.

No studies were identified where decision-making or policy at the meso-level (regional, provincial or state level) was the target.

### Limitations and challenges associated with using system dynamics for environmental health decision-making

Twelve reports referred to limitations and challenges associated with using system dynamics modelling within the context of the problem being addressed. For the purpose of this review, only limitations and challenges related to the process of system dynamics modelling or the overall functionality of system dynamics models were analysed. Limitations for the purposes of this review were grouped into three categories: limitations related to those involved in the modelling process, technical limitations of system dynamics, and limitations related to the application of the results to decision-making (Table 5). Note that none of the limitations or challenges identified by the authors appeared to be related specifically to the application of system dynamics to environmental health problems, but rather to the application of system dynamics in general.

**Table 5:** Limitations and challenges associated with system dynamics modelling

Limitation/Challenge	Number of studies [reference]
User-related	
Participants needed subject-matter knowledge and familiarity with system dynamics to meaningfully participate in the modelling process	3 [33, 39, 40]
Those using the model but not closely involved in the model-making process struggled to understand and trust the model and its results	2 [30, 36]
Significant commitment and time investment needed by participants	1 [37]
Complexity of the system dynamics model may make it difficult for users to understand the details of the model and this may increase the perception that the problem is so complex that it is not feasible to tackle	2 [33, 36]
Potential unwillingness of participants to have their perception / beliefs about the problem (otherwise known as mental models) challenged	1 [37]
Technical	
The inclusion of subjective variables whose behaviour may be influenced by interpretation-bias	1 [38]
The inherent uncertainty regarding variables and causal structures of complex problems, resulting key variables being unintentionally omitted from the model	1 [32]
The inclusion of parameters whose values are unknown and cannot reasonably be estimated	1 [32]
The creation of fully endogenous models of large and complex problems can result in huge ‘data hungry’ models	1 [32]
The accuracy and comprehensiveness of the model depends heavily on the inclusion of an appropriate mix of stakeholders	1 [35]
the complexity of the end product may result in end-users requiring a model guide in order to effectively use the model	1 [36]
The model’s output did not provide specific directions for end-users, but rather showed possible future trends and relative magnitudes of impact	2 [38, 40]
Application-related	
System-wide changes are difficult to implement given the often ‘siloed’ nature of public governance structures	2 [41, 43]
There is potential for incompatibility between the timescale of political and public decision-making and system dynamics model-building, which results in rushed and over-simplified model	1 [37]
Obtaining decision-maker buy-in is difficult due to the disparity that exists between system dynamics’ goal of identifying sources of long-term success, and political processes which focuses primarily on short-term goals and outcomes	1 [37]

## Discussion

To our knowledge, a review of the applications of system dynamics to environmental health decision-making has not been previously conducted. Our findings suggest that the applications of system dynamics to environmental health decision-making is limited given the paucity of reporting, however the applications that were found vary across many different sectors. Our review further suggested that the use of system dynamics in this context represents an emerging field of research and practice, with the number of studies published increasing considerably in the last five years. While many of the reported studies occurred within the public health or health service sectors, there were a variety of non-health-specific sectors using system dynamics to model both environmental and health factors.

The 1986 Ottawa Charter for Health Promotion recognised that the promotion of health cannot be done by the health-sector alone, and that the success of health-promoting activities, such as those occurring within the field of environmental health, depend on the successful collaboration and support of many different sectors [44]. Our review strongly suggests that system dynamics can be used as a method to facilitate the integration of health into policy and decision-making processes in non-health sectors.

System dynamics not only provides direct decision-support for policy analysis, but also provides a means for understanding problems, which in turn informs the way decisions-makers navigate complex decision-making processes. This is consistent with the way in which system dynamics has been used to address complex problems in other fields such as health policy [45], social care [46], strategic management [47], and transportation [48].

System dynamics has been used to inform decision-making at either the micro or macro-level, with limited application targeted at decision-making across multiple levels. While this is potentially due to disconnects between decision-making processes at the micro- and macro-level, it does point to a potential missed opportunity. Complex environmental health problems do not respect jurisdictional boundaries, and can rarely be addressed at only the local or national level. While likely not possible in all situations, the URAdapt project outlined in Raschid-Sally, et al. [43] demonstrates the potential for system dynamics to be used to address environmental health problems at multiple decision-making levels.

None of the reports included in this review spoke of the effectiveness of the policies or decisions that resulted from the use of system dynamics. While this is not surprising given the recentness of their publication and the time delays between the implementation of decisions and their effects, it does indicate a gap in the literature. Evaluations of the effectiveness of decisions or policies developed based on system dynamics models will help researchers and decision-makers evaluate the merit of using system dynamics to address complex problems.

Multiple limitations and challenges associated with the application of system dynamics were discussed in the reports reviewed, but none were specifically directed at its feasibility for application to environmental health problems. Many of the limitations, such as the level of complexity of the models and a lack of participant openness to challenge their mental models, were consistent with applications of system dynamics in other fields [49, 50].

It is worth noting that some of the identified limitations, such as potential unwillingness of participants to have their mental models challenged, are not necessarily limitations of system dynamics itself, but rather a limitation of the way it is used. While using system dynamics in a participatory learning process increases the chances that participants and stakeholders will critically reflect on their own mental models, there are no guarantees that system dynamics, or any other policy analysis process, can change deeply-held views and positions. These issues are not insurmountable, but require further research and action from both the system dynamics scientists and practitioner community and the wider policy and governance communities. As systems approaches become more widespread and integrated into the way current and future decision-makers think about complex problems, the greater the chance that tools like system dynamics will find a receptive audience.

The number of studies that met the inclusion criteria but were ultimately excluded from the review suggests a missing link when it comes to the application of system dynamics to environmental health decision-making. For instance, reports by McClure et al. [20] that examined the relationships among transportation, economic development, land use, and population health; and by Pedercini, et al. [21] that examined the costs and benefits of various malaria control interventions, demonstrated interesting and novel applications of system dynamics in environmental health, but did not link the outcomes of their system dynamics models to a decision-making process. This suggests a gap between basic and applied system dynamics in environmental health and decision-making and policy. The water security model reported by El Sawah et al. [51] and the waste management model reported by Stave’s [52] suggest how system dynamics could inform decision-making within the field of environmental health, but these applications lacked a health or health hazard component in their model. This also reflects a disconnect between the fields of environmental management and human health. System dynamics does have significant potential to bridge the gap between environmental management and human health, however this needs further demonstration.

### Limitations of the review

This review has several limitations that must be recognised when interpreting the results. First, it identified and summarized only 15 publications, all of which were varied in their rationale and scope. This heterogeneity suggests that while key common elements of the studies could be identified, the complexity of the individual studies were not fully captured. Second, the limited number of reports identified prevents a comprehensive assessment of the benefits and limitations of applying system dynamics to environmental health issues. More applications of system dynamics in this context is called for. Third, the multi-disciplinary nature of environmental health may have caused us to miss some relevant reports. For example, work occurring in sectors such as environmental management, waste management and urban planning – although often not so intended - will directly or indirectly influence human health. It is possible that some authors may not have labelled their work as being related to ‘environmental health’, making it difficult to capture it in reviews such as this one. Similarly, authors describing applications of system dynamics modelling may have used different terminology to describe their modelling approach, which would have caused their work to be missed by this scoping review. Finally, this review relied on reports being in the public domain. While a systematic search of the scientific and grey literature is likely to capture most reports originating from academia, it is likely that there are reports based on system dynamics applications that have not been published.

## Conclusion

In a world of rapidly changing environments, environmental health decisions and policies must be made regardless of the complexity of environmental health problems and significant uncertainties about the future. It is crucial that we move past discussing the general merits of a systems approach to addressing environmental health problems, to determining how best to apply systems tools and methods to existing environmental health problems.

Our review suggests that system dynamics is being applied sparingly, but increasingly within the field of environmental health. The method has significant potential when it comes to assisting decision-makers understand and analyse complex environmental health issues, as well predict the outcomes environmental health decisions and policies. System dynamics is currently being used in multiple different sectors to provide direct and indirect decision-support for population health policy and decision-making processes. Despite this, the contribution of system dynamics to environmental health policy remains limited. Current decision-making and governance structures in many public organisations are not designed to integrate the multi-sectoral policy advice that system dynamics can provide. Therefore, the full potential of system dynamics to change the way in which environmental health problems are managed cannot be realised unless the decision-making and governance structures of public organisations are collaborative and coordinated.

## References

[1] Keune H (2012). Critical complexity in environmental health practice: simplify and complexify. Environmental health : a global access science source.

[2] Tarkowski SM (2007). Environmental health research in Europe: bibliometric analysis. Eur J Pub Health.

[3] Kreuter MW, De Rosa C, Howze EH, Baldwin GT: Understanding wicked problems: a key to advancing environmental health promotion. Health education & behavior : the official publication of the Society for Public Health Education 2004, 31(4):441–454. 10.1177/1090198104265597 15296628

[4] Forrester JW: Urban dynamics, vol. 114: mIt press Cambridge; 1969.

[5] Smith AH, Lingas EO, Rahman M (2000). Contamination of drinking-water by arsenic in Bangladesh: a public health emergency. Bull World Health Organ.

[6] Milner J, Shrubsole C, Das P, Jones B, Ridley I, Chalabi Z, Hamilton I, Armstrong B, Davies M, Wilkinson P. Home energy efficiency and radon related risk of lung cancer: modelling study. BMJ. 2014;348 10.1136/bmj.f7493 PMC3898159 24415631

[7] Sterman JD (2006). Learning from evidence in a complex world. Am J Public Health.

[8] Atkinson J-A, Page A, Wells R, Milat A, Wilson A (2015). A modelling tool for policy analysis to support the design of efficient and effective policy responses for complex public health problems. Implement Sci.

[9] Forrester JW (1958). Industrial dynamics - a major breakthrough for decision makers. Harv Bus Rev.

[10] Sterman JD, Fiddaman T, Franck T, Jones A, McCauley S, Rice P, Sawin E, Siegel L (2013). Management flight simulators to support climate negotiations. Environ Model Softw.

[11] Dangerfield BC (1999). System dynamics applications to European health care issues. J Oper Res Soc.

[12] Minegishi S, Thiel D (2000). System dynamics modeling and simulation of a particular food supply chain. Simulation Practice and Theory.

[13] Bakken BT, Gilljam M (2003). Dynamic intuition in military command and control: why it is important, and how it should be developed. Cogn Tech Work.

[14] Homer JB, Hirsch GB (2006). System dynamics modeling for public health: background and opportunities. Am J Public Health.

[15] Bosch O, Maani K, Smith C (2007). Systems thinking - language of complexity for scientists and managers. Improving the triple bottom line returns from small-scale forestry: 18–21 June 2007.

[16] Sterman JD (2000). Business dynamics: systems thinking and modeling for a complex world, vol. 19: Irwin.

[17] Cox LA, Ricci PF (2008). Causal regulations vs. political will: why human zoonotic infections increase despite precautionary bans on animal antibiotics. Environ Int.

[18] Shahgholian K (2009). A Dynamic Model of air pollution, health, and population growth using system dynamics: a study on Tehran-Iran (with computer simulation by the software Vensim). World Acad Sci Eng Technol.

[19] Macmillan A, Connor J, Witten K, Kearns R, Rees D, Woodward A (2014). The societal costs and benefits of commuter bicycling: simulating the effects of specific policies using system dynamics modeling. Environ Health Perspect.

[20] McClure RJ, Adriazola-Steil C, Mulvihill C, Fitzharris M, Salmon P, Bonnington CP, Stevenson M (2015). Simulating the dynamic effect of land use and transport policies on the health of populations. Am J Public Health.

[21] Pedercini M, Movilla Blanco S, Kopainsky B (2011). Application of the malaria management model to the analysis of costs and benefits of DDT versus non-DDT malaria control. PLoS One.

[22] Galea S, Riddle M, Kaplan GA (2010). Causal thinking and complex system approaches in epidemiology. Int J Epidemiol.

[23] Luke DA, Stamatakis KA (2012). Systems science methods in public health: dynamics, networks, and agents. Annu Rev Public Health.

[24] Diez Roux AV (2011). Complex systems thinking and current impasses in health disparities research. Am J Public Health.

[25] Peters MD, Godfrey CM, Khalil H, McInerney P, Parker D, Soares CB (2015). Guidance for conducting systematic scoping reviews. International journal of evidence-based healthcare.

[26] Arksey H, O'Malley L (2005). Scoping studies: towards a methodological framework. Int J Soc Res Methodol.

[27] Levac D, Colquhoun H, O'Brien KK (2010). Scoping studies: advancing the methodology. Implement Sci.

[28] Adam T (2014). Advancing the application of systems thinking in health. Health Research Policy and Systems.

[29] Birnbaum LS, Jung P (2010). Evolution in environmental health: incorporating the infectious disease paradigm. Environ Health Perspect.

[30] Kenealy T, Rees D, Sheridan N, Moffitt A, Tibby S, Homer J (2012). A ‘whole of system’ approach to compare options for CVD interventions in counties Manukau. Aust N Z J Public Health.

[31] Newman J, Martin L, Velasco MA, Fantini A-M: A system dynamics approach to monitoring and evaluation at the country level: An Application to the Evaluation of Malaria-Control Programs in Bolivia. In: 5th Biennial World Bank Conference on Evaluation and Development: 15–16 July 2003; Washington DC: World Bank; 2003.

[32] Lane D (2014). Modelling the foodborne transmission mechanisms for norovirus.

[33] Brennan LK, Sabounchi NS, Kemner AL, Hovmand P (2015). Systems thinking in 49 communities related to healthy eating, active living, and childhood obesity. Journal of public health management and practice : JPHMP.

[34] Feola G, Gallati JA, Binder CR (2012). Exploring behavioural change through an agent-oriented system dynamics model: the use of personal protective equipment among pesticide applicators in Colombia. Syst Dyn Rev.

[35] Macmillan A, Davies M, Shrubsole C, Luxford N, May N, Chiu LF, Trutnevyte E, Bobrova Y, Chalabi Z (2016). Integrated decision-making about housing, energy and wellbeing: a qualitative system dynamics model. Environ Health.

[36] Olabisi LS, Levine R, Cameron L, Beaulac M, Wahl R, Blythe S. A modeling framework for informing decision maker response to extreme heat events in Michigan under climate change. In: Brown DB, editor. 2011 project reports. D; Briley, L: Great Lakes Integrated Sciences and Assessments (GLISA) Center; 2012.

[37] Stave KA (2002). Using system dynamics to improve public participation in environmental decisions. Syst Dyn Rev.

[38] Mahamoud A, Roche B, Homer J (1982). Modelling the social determinants of health and simulating short-term and long-term intervention impacts for the City of Toronto, Canada. Soc Sci Med.

[39] Loyo HK, Batcher C, Wile K, Huang P, Orenstein D, Milstein B (2013). From model to action: using a system dynamics model of chronic disease risks to align community action. Health Promot Pract.

[40] Kolling J, Cox L, Flanders N, Proctor A, Tanners N, Bassi A, Araujo R (2016). A system dynamics model for integrated decision making: the Durham-Orange light rail project. In.

[41] Chen CH, Liu WL, Liaw SL, Yu CH (2005). Development of a dynamic strategy planning theory and system for Sustainable River basin land use management. Sci Total Environ.

[42] Stave KA, Dwyer M: Lessons from LUTAQ: Building systems thinking capacity into land use, transportation, and air quality planning in Las Vegas, Nevada In: 24th International Conference of the System Dynamics Society: 23–27 July 2006. Nijmegen; 2006.

[43] Raschid-Sally L, Moges S, Sahilu G, Bayrau A, Amisigo B, Akoto-Danso EK. Managing water at the urban-rural Interface: the key to climate change resilient cities (URAdapt). International Water Management Institute; 2013.

[44] World Health Organization, Health and Welfare Canada, Canadian Public Health Association: Ottawa Charter for Health Promotion: An international conference on health promotion - the move towards a new public health, Nov. 17–21. Ottawa: World Health Organization; 1986.

[45] Atkinson J-A, Wells R, Page A, Dominello A, Haines M, Wilson A. Applications of system dynamics modelling to support health policy. Public Health Res Pract. 2015;25(3):e2531531 10.17061/phrp2531531 26243490

[46] McKelvie D (2013). Modelling social care complexity: the potential of system dynamics.

[47] Cosenz F, Noto G. Applying system dynamics modelling to strategic management: a literature review. Syst Res Behav Sci. 2016;

[48] Shepherd SP (2014). A review of system dynamics models applied in transportation. Transportmetrica B: Transport Dynamics.

[49] Rouwette EAJA, JAM V, Tv M (2002). Group model building effectiveness: a review of assessment studies. Syst Dyn Rev.

[50] Caulfield CW, Ma SP (2001). A case for systems thinking and system dynamics. IEEE international conference on systems, man and cybernetics.

[51] El Sawah S, McLucas A, Mazanov J. Communication about Water Management in the Australian Capital Territory: A System Dynamics Modelling Approach In: 27th International Conference of the System Dynamics Society: July 26–30 2009. Albuquerque, New Mexico; 2009.

[52] Stave KA (2008). Zero waste by 2030: a system dynamics simulation tool for stakeholder involvement in Los Angeles‘ solid waste planning initiative. 26th international conference of the system dynamics society: 20–24 July.

[53] Pasqualini D, Witkowski M, Klare P, Patelli P, Cleland C. A Model for a Water Potable Distribution System and its Impacts resulting from a Water Contamination Scenario. In: International Conference of System Dynamics Society. The Netherland; 2006.

